# Probiotics for infantile colic: a systematic review

**DOI:** 10.1186/1471-2431-13-186

**Published:** 2013-11-15

**Authors:** Jasim Anabrees, Flavia Indrio, Bosco Paes, Khalid AlFaleh

**Affiliations:** 1 Neonatal Care, Sulaiman Al Habib Medical Group, Arrayan Hospital, P.O. Box 272069, Riyadh, 11352, Saudi Arabia; 2 Department of Pediatrics, University of Bari, Bari, Italy; 3 Department of Pediatrics (Neonatal Division), McMaster University, Hamilton, Ontario, Canada; 4 Department of Pediatrics, King Saud University, Riyadh, Saudi Arabia

**Keywords:** Infantile colic, Probiotics, Systematic review, Lactobacillus reuteri

## Abstract

**Background:**

Infantile colic is a common paediatric condition which causes significant parental distress. Increased intestinal coliform colonization in addition to alteration in Lactobacillus abundance and distribution may play an important role in its pathogenesis.

The objectives of this systematic review are to evaluate the efficacy of probiotic supplementation in the reduction of crying time and successful treatment of infantile colic.

**Methods:**

Literature searches were conducted of MEDLINE, EMBASE and the Cochrane Central Register of Controlled Trials. Only randomized controlled trials enrolling term, healthy infants with colic were included. A meta-analysis of included trials was performed utilizing the Cochrane Collaboration methodology.

**Results:**

Three trials that enrolled 220 breastfed infants met inclusion criteria, of which 209 infants were available for analysis. Two of the studies were assessed as good quality. *Lactobacillus reuteri (*strains-American Type Culture Collection Strain 55730 and DSM 17 938) was the only species utilized in the therapeutic intervention. Two of the trials were industry funded. Probiotic supplementation compared to simethicone or placebo significantly and progressively shortened crying times to 7 days reaching a plateau at three weeks post initiation of therapy [mean difference −56.03 minutes; 95% CI (−59.92, -52.15)]. Similarly, probiotics compared to placebo significantly increased the treatment success of infantile colic with a relative risk (RR) of 0.06; 95% CI (0.01, 0.25) and a number needed to treat of 2.

**Conclusions:**

Although *L. reuteri* may be effective as a treatment strategy for crying in exclusively breastfed infants with colic, the evidence supporting probiotic use for the treatment of infant colic or crying in formula-fed infants remains unresolved. Results from larger rigorously designed studies will help draw more definitive conclusions.

## Background

Infantile colic is a common problem in healthy thriving infants that is associated with excessive crying over a regular period during the day and is sustained for the first few months of life [1,2]. The condition has been historically described as irritable or compulsive crying or paroxysmal fussing with a multifactorial etiology [3]. Although it affects 5% -19% of young infants, [2,4,5] it remains a frustrating problem for parents and care givers because it is difficult to treat and may result in significant psychosocial consequences. A number of cross sectional studies report significant links between infantile colic and maternal depression and child abuse [6-11].

Despite forty years of research, the etiology of infantile colic remains elusive. The current literature suggests several causative mechanisms such as behavioral, food allergy and hypersensitivity, immaturity of gut function and dysmotility [12-14]. Of note, Shenassa et al. through a comprehensive review of 5 studies identified a possible link between maternal smoking and infantile colic which may be mediated through increased plasma and intestinal motilin levels [15]. Recently, the composition of intestinal microbiota has been addressed as an independent risk factor for infantile colic [16-18]. Studies indicate that inadequate lactobacilli in the first few months of life may affect intestinal fatty acid profile favoring the development of infantile colic [16,17]. Coliform bacteria have also been found more abundantly in colicky infants and it is speculated that altering the intestinal microbiota composition may positively influence the management of affected infants [19]. In practice, the only probiotic used for infantile colic is *Lactobacillus reuteri* (strains-American Type Culture Collection Strain 55730 or DSM 17 938). However, other Lactobacillus species such as *L.delbrueckii* subsp.*delbrueckii* DSM 20074 and *L. plantarum* MB 456 have proven inhibitory activity against gas-forming coliforms and may play a significant role in the management of infantile colic [20]. Similarly, Aloisio et al. evaluated four Bifidobacterium strains, namely, *B. breve* B632 (DSM 24706), B2274 (DSM 24707), B7840 (DSM 24708), and *B. longum* subsp. *longum* B1975 (DSM 24709), and found that they may be potentially useful for the treatment of infantile colic or as a preventive strategy for infantile bacterial-related diarrhea [21]. However, exploratory clinical trials investigating both the safety and efficacy of probiotics incorporating these species are yet to be conducted.

The objectives of this systematic review are to evaluate the efficacy of probiotic supplementation in the reduction of crying time and successful treatment of infantile colic.

## Methods

### Search strategy

Eligible studies were identified from OVID MEDLINE – National Library of Medicine [January 1966 to September 2012] using the following subject MeSH headings and text word terms: neonate(s), newborn(s), infant(s), probiotics, lactobacillus, bifidobacterium, colic; publication type was limited to controlled trials. No language restriction was applied. Other databases were also searched including: EMBASE (January 1980 to September 2012) the Cochrane Central Register of Controlled Trials (CENTRAL, the Cochrane Library, Issue 9, 2012). Additional citations were sought using references in articles retrieved from searches. Content experts were contacted to identify unpublished and ongoing studies.

### Study selection

We included all randomized or quasi-randomized controlled trials that compared probiotics (any dose or composition) to placebo, control or other forms of treatment in healthy full term infants with infantile colic who were less than 4 months of age. All definitions of infantile colic were deemed acceptable. We considered articles in any language as long as there was an abstract in English indicating content.

### Data extraction

Retrieved articles were assessed for eligibility, and two reviewers independently abstracted descriptive data on the subjects, type of intervention, infants allocated as control, outcomes and methodological quality of the articles. Discrepancies were resolved by discussion and consensus. Where data were incomplete, the principal investigator of the primary study was contacted for further information and clarification.

### Methodological quality of the studies

Standard methods of the Cochrane Collaboration [22] were used to assess the methodological quality of included trials. For each trial, information was sought regarding the method of randomization, allocation concealment, blinding, and completeness of follow up and on reported outcomes of all infants enrolled in the trial. The methodological details of the studies were extracted from published data and by contacting the primary author where possible.

### Data synthesis

The primary outcome was treatment success, defined as the percentage of children who achieved a reduction in the daily average crying time >50%. The secondary outcomes were duration of crying (minutes per day) and adverse events related to probiotic supplementation. For dichotomous outcomes, relative risk (RR) and its associated confidence interval were calculated. For continuous outcomes, treatment effect was expressed as mean difference and its calculated standard deviation. Meta-analysis of pooled data was performed using a fixed effects model with the assumption that L. *reuteri* DSM 17938 and L. *reuteri* ATCC 55730 are bioequivalent and the added recognition that the comparison groups were simethicone (one trial) or placebo (two trials). Review Manager (RevMan), Version 5.2 software was used for statistical analysis. A subgroup analysis was planned a priori to investigate the effect of probiotics in subjects with a positive family history of atopy, and additionally on different strains of probiotics utilized. Heterogeneity was defined as a significant test when the p value was < 0.1 and/or if similar differences were identified in treatment effects across studies. Tests for between-study heterogeneity (including the I^2^ test) were performed.

Since included studies expressed their primary outcome (crying times) as median (range or interquartile range), in order to statistically pool the data and perform a meta-analysis, this outcome was converted into mean (standard deviation) as recommended by Hozo et al. [23].

## Results

**Figure 1:**
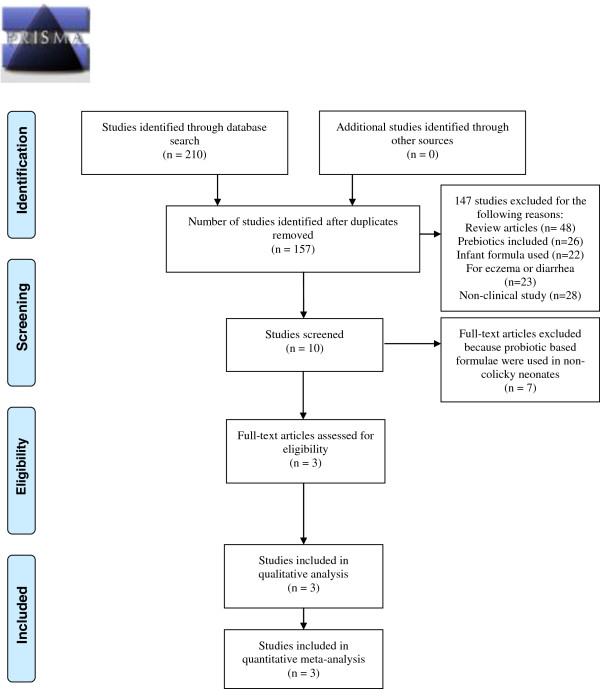
PRISMA flow diagram 2009 of included studies.

A total of 10 potentially relevant citations were obtained through our primary search strategy (Figure 1). Seven studies were excluded because the investigators used probiotic based formulae in non-colicky neonates, which undermined the primary objectives of the meta-analysis. Three trials met our inclusion criteria [24-26]. Although Szajewska et al. [26] planned an inclusion criterion of infants aged less than five months, the actual maximum age at enrolment was 81 days. Characteristics of the included trials are summarized in Table 1. Three ongoing trials are summarized in Table 2, but the data since incomplete were not included in this review.

**Table 1:** Characteristics of trials included in the analysis

**Study/year/reference**	**Description/study design**	**Birth weight and age at enrolment**	**Probiotic agent(s)**	**Dosage and duration**	**Control arm**	**Primary outcome**
Savino/2007 [24]	April 2004 - May 2005.	Birth weight 2500-4000 g and aged 21–90 days	*L. reuteri* (American Type Culture Collection Strain 55730)	10^8^ colony-forming units in 5 drops for 28 days	Simethicone	A reduction of average crying time to less than 3 hours a day on day 28.
	90 exclusively breastfed infants with a diagnosis of infantile colic.					
	Recruited in the Department of Pediatric and Adolescence Science (Regina Margherita Children Hospital, Turin, Italy)					
	Open prospective randomized study.					
Savino/2010 [25]	March 2008 and August 2009.	Birth weight 2500–4000 g and aged 2–16 weeks	*L. reuteri* DSM 17938	10^8^ colony-forming units in 5 drops, once a day, 30 minutes before the feed in the morning, for 21 days	Placebo	A reduction of average crying time to less than 3 hours a day on day 21.
	50 exclusively breastfed infants were recruited from general pediatricians and outpatients at the Department of Pediatrics, University of Turin (Regina Margherita Children Hospital)					
	A Randomized, Double-Blind, Placebo-Controlled Trial					
Szajewska/2013 [26]	January 2010 and December 2011.	Full term infants aged <5 months	*L. reuteri* DSM 17938	10^8^ colony-forming units in 5 drops, orally, once a day, for 21 days	Placebo	The percentage of children achieving a reduction in the daily average crying time more than 50% and the duration of crying at 7, 14, 21, and 28 days after randomization.
	80 exclusively or predominantly (>50%) breastfed infants					
	Family primary care practice in Warsaw, Poland					
	A Randomized, Double-Blind, Placebo-Controlled Trial					

Colic in all the trials was defined as crying episodes lasting ≥3 hours/day and occurring at least 3 days/week within 7 days prior to enrollment.

**Table 2:** The ongoing trials of probiotics and infant colic

**No**	**Study**	**Inclusion and exclusion criteria**	**Primary outcome**	**Estimated enrollment**	**Arms**
1	Effect of *L. rhamnosus* GG (LGG) on Infant Colic	Inclusion Criteria:	Crying times of infants	60	Experimental: Nutramigen Lipil with Enflora
		Sixty healthy full-term colicky infants (gestational age 32–41 weeks)			
					Control: Nutramigen A + Hypoallergenic formula without lactobacilli
		Exclusion Criteria:			
		Chronic lung disease,			
		Diarrhea (stools that take the shape of a container > 5x daily)			
		Fever			
2	Control of Colic in Infants by Dietary Supplementation with the Probiotic *L. reuteri*	Inclusion Criteria:	Reduction of daily average crying time to less than 3 hours from baseline	50	Experimental: *L. reuteri*
		Infants aged between 14–60 days			
					Control: Not clear
		Breast fed, exclusively during length of trial			
		Diagnosis of infantile colic according to Wessel’s criteria			
		Debut of colic symptoms 6 ± 1 days before randomization			
		Gestational age between 37–42 weeks			
		Apgar score higher than 7 at 5 minutes			
		Mothers willing to follow a cow milk-free diet during the study period			
		Written informed consent and stated availability throughout the study period			
		Exclusion Criteria:			
		Major chronic disease			
		Gastrointestinal disease but controlled gastroesophageal reflux disease			
		Administration of antibiotics the week before randomization			
		Administration of probiotics the week before randomization			
		Participation in other clinical trials			
3	Baby Biotics randomised controlled trial	Inclusion criteria	Infant crying/fussing time	160	Experimental: L. reuteri DSM 17938.
		Infant colic as defined by the modified Wessel’s criteria			
			(min/day)		
		Less than 3 months			Control: maltodextrose
		Greater than 36 weeks gestation at birth			
		Birth weight of more than 2500 g.			
		Exclusion criteria			
		Failure to thrive			
		Major medical problems			
		Taking solids, antibiotics or *L. reuteri* and, if breastfeeding, mother taking			
		*L. reuteri* at the time of study commencement;			
		Cow’s milk protein allergy			
		Caregiver has insufficient English to understand informed consent and complete questionnaires.			

Overall, 140 infants were exclusively breastfed [24,25] while Szajewska et al. additionally reported that 80 of the infants in their trial were exclusively or predominantly (>50%) breastfed [26]. In general, included trials had a low risk of bias (Table 3). A total of 209 healthy infants were enrolled across the three studies and most of the infants were exclusively breast fed. All of the clinical trials utilized the same probiotic species (*Lactobacillus reuteri; strains-American Type Culture Collection Strain 55730 or DSM 17 938*) with identical daily doses. One study evaluated the efficacy of probiotic supplementation against simethicone [24]. None of the included studies reported any adverse side effects of supplementation.

**Table 3:** The quality and risk of bias in the trials included in the analysis

**Study/year/reference**	**Random sequence generation (selection bias)**	**Allocation concealment (selection bias)**	**Blinding of participants and personnel (performance bias)**	**Blinding of outcome assessment (detection bias)**	**Incomplete outcome data (attrition bias)**	**Selective reporting (reporting bias)**	**Other bias**
Savino/2007 [24]	Low risk	Low risk	High risk	Low risk	Low risk	Low risk	Low risk
Savino/2010 [25]	Low risk	Unclear risk	Low risk	Low risk	Low risk	Low risk	Low risk
Szajewska/2013 [26]	Low risk	Low risk	Low risk	Low risk	Low risk	Low risk	Low risk

### Effect of L. reuteri on crying time

**Figure 2:**
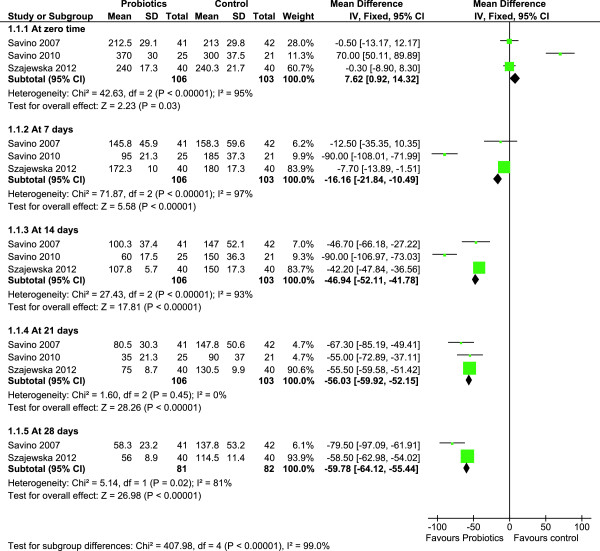
**Forest plot of L. *reuteri *** **ATCC 55730 and L. *reuteri *** **DSM 17938 versus control effect in decreasing mean crying times (min) over 28 days.**

The effect of L. reuteri on crying time was compared to simethicone or placebo. Data on crying time were reported by all three trials as a primary outcome and involved 209 infants. At seven days after initiation of treatment, infants in the probiotic group had a significantly shorter crying time. The crying time at 7 days was significant only in the fixed effects model, but was insignificant in the random effects model. However, the treatment effect was continuous and stabilized at three weeks following the initiation of therapy. Probiotics decreased crying times by almost one hour [mean difference −56.03 minutes; 95% CI (−59.92, -52.15)] (Figure 2). In order to reduce heterogeneity and the potential effect of simethicone, a sensitivity analysis on only the double-blind, placebo-controlled, trials [24,26] was conducted. The heterogeneity remained unaltered with a strikingly similar reduction in crying time at 21 days [mean difference −55.48 minutes; 95% CI (−59.46, -51.49)].

### Effect of L. reuteri on overall response rate

**Figure 3:**
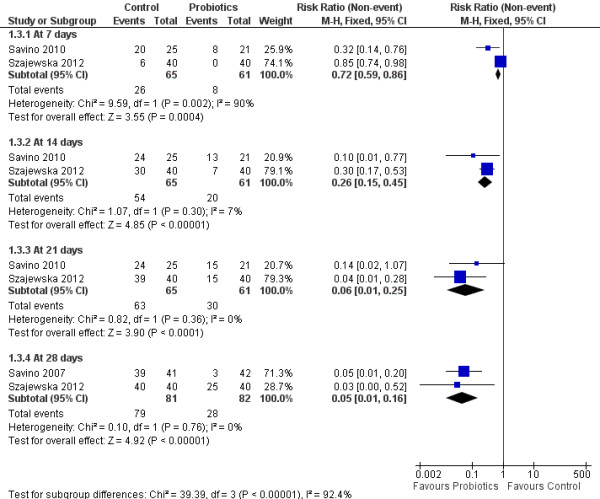
**Forest plot of L. *reuteri *** **ATCC 55730 and L. *reuteri *** **DSM 17938 versus control effect in improving infantile colic treatment success rate over 28 days.**

The overall response rate of L. *reuteri* was compared to simethicone or placebo. Responders (or treatment success) was defined as the percentage of infants achieving a reduction in the daily average crying time of more than fifty percent. The response rate was reported in two of the trials at each assessment interval. Savino et al. reported the response rate at 28 days only [24]. A progressive, statistically significant response was noted starting at 7 days [25] after initiation of therapy (Figure 3). The effect was maximal at 21 days following the commencement of treatment, with a relative risk (RR) of 0.06; 95% CI (0.01, 0.25) and a number needed to treat (NNT) of 2. Of note, a similar progressive improvement was also evident in the control subjects; however, the positive effect was more pronounced in the probiotic group.

### Effect with a history of atopy

The impact of atopy was documented in only one study [26]. A concomitant history of atopy did not alter the efficacy of probiotics in treated infants.

## Discussion

We report the first systematic review of randomized trials addressing the efficacy of probiotics in infantile colic. A significant effect of *L. reuteri* supplementation (strains-American Type Culture Collection Strain 55730 and DSM 17 938) in shortening crying times and improvement in response rate was noted. This positive response was progressive with time and had its peak and plateau at three weeks after initiation of therapy. A similar positive effect was noted in the control group, which could be explained, by the natural history of infantile colic or a placebo effect [22,23]. However, the effect in the probiotic group was more pronounced. It is important to note that one of the strains (*L. reuteri* ATCC 55730) used in one of the included trials [24] was found to carry potentially transferable resistance traits for tetracycline and lincomycin in adults [27] and was replaced in subsequent studies by *L. reuteri* DSM 17938, a daughter strain that retained the original probiotic characteristics [28,29].

Our systematic review included currently available, high quality studies. However; our overall conclusion is compromised by the small number of enrolled infants despite the common prevalence of infantile colic, and the heterogeneity of included studies that assessed crying time. This heterogeneity can be explained by the clear imbalance of both groups at baseline, in the study reported by Savino et al. [25]. The probiotic group started with significantly longer crying times that quickly moved to a profoundly positive benefit after 7 days of treatment. Most of the included subjects were exclusively breast fed which limits the generalizability of the findings to formula fed infants. However, the incidence of colic in breast and bottle fed infants is similar [30] because the pathogenesis of the disorder is likely multifactorial and the use of probiotics may be influential in the treatment of colic by altering faecal microbiota and gut inflammation irrespective of feeding patterns [16-19,31].

The earlier trial by Savino et al. [24] involved the use of simethicone in the placebo arm. This may have decreased the magnitude of the effect of probiotics unless the effect of simethicone was not indifferent to placebo. The sensitivity analyses confirmed that the inclusion of the simethicone treated patients in this study did not significantly impact heterogeneity or the crying time at 21 days. One well recognized limitation of all studies on infant colic is the need for a more objective way of measuring duration of crying rather than relying on the parents’ compliance to establish this outcome. Computer recordings of crying episodes comprise a more concrete form of assessment and should be considered for future studies. Two of the included trials were supported by the manufacturer of the probiotic strain under study, which raises the possibility of bias. However, the likelihood is small since the trials were fully investigator-initiated and data controlled with transparent disclosure of potential conflicts of interest by the respective authors [32,33].

Recently few studies have addressed the role of changing intestinal microbiota in the pathogenesis of colic. Colicky infants were found to have increased colonization by coliforms especially E.coli and decreased and altered colonization patterns by lactobacillus species [17-19]. Furthermore, *L. reuteri* also exerts an antimicrobial effect against enteric pathogens which may induce an immunologic response [34-36]. Immune modulation could also play a role in the efficacy of probiotics in infantile colic as it may represent the first sign of food hypersensitivity [37]. Since probiotic supplementation appears to require time to exert an effect in colicky infants, it would be interesting to evaluate its efficacy as a prophylactic treatment after birth. It’s unclear whether a similar or cumulative effect would be observed if other formulations of probiotic bacteria are utilized either alone or in combination with the same strains of *L. reuteri*.

## Conclusions

Our review supports the beneficial effects of probiotic supplementation in infantile colic in predominantly breast fed infants. *L. reuteri (strains-American Type Culture Collection Strain 55730 and DSM 17 938)* significantly decreased the rate (minutes/day) of crying and no short term safety concerns were identified. However, all three included studies demonstrate a positive outcome which may be a reflection of the relatively small, combined, sample size that overshadows the true effect which may be realized in a single, large-scale, multicenter, randomized trial. More independent studies are still required in diverse ethnic populations, especially in formula fed infants, [38] prior to adopting a change in practice.

## Abbreviations

CI: Confidence interval; NNT: Number needed to treat; RR: Relative risk.

## Competing interests

The authors declare that they have no competing interests.

## Authors’ contributions

JA: Conceptualized the study design, protocol development, inclusion selection, quality assessment and statistical analysis, and drafted the initial manuscript. FI: Was involved in the study conception and design, oversaw the protocol development and data interpretation and participated in the manuscript preparation. BP: Critically reviewed and amended the manuscript and circulated the final version for approval and submission. KA: Played a major role in the study conception, design, protocol development, inclusion selection, quality assessment and statistical analysis and worked collaboratively on the draft of the initial manuscript. All authors read and approved the final manuscript.

## Pre-publication history

The pre-publication history for this paper can be accessed here:

http://www.biomedcentral.com/1471-2431/13/186/prepub

## References

[B1] Illingworth RS Infantile colic revisited Arch Dis Child 1985 60 981 985 3840672 10.1136/adc.60.10.981 PMC1777482

[B2] Lucassen PL Assendelft WJ van Eijk JT Systematic review of the occurrence of infantile colic in the community Arch Dis Child 2001 84 398 403 11316682 10.1136/adc.84.5.398 PMC1718751

[B3] Wessel MA Cobb JC Jackson EB Harris GS Jr Detwiler AC Paroxysmal fussing in infancy, sometimes called colic Pediatrics 1954 14 421 435 13214956

[B4] Castro-Rodríguez JA Stern DA Halonen M Relation between infantile colic and asthma/atopy: a prospective study in an unselected population Pediatrics 2001 108 878 882 11581439 10.1542/peds.108.4.878

[B5] Gelfand AA Thomas KC Goadsby PJ Before the headache: infant colic as an early life expression of migraine Neurology 2012 79 1392 1396 22972642 10.1212/WNL.0b013e31826c1b7b PMC4098946

[B6] Vik T Grote V Escribano J European Childhood Obesity Trial Study Group Infantile colic, prolonged crying and maternal postnatal depression Acta Paediatr 2009 98 1344 1348 19432839 10.1111/j.1651-2227.2009.01317.x

[B7] Akman I Kuscu K Ozdemir N Mothers’ postpartum psychological adjustment and infantile colic Arch Dis Child 2006 91 417 419 16452109 10.1136/adc.2005.083790 PMC2082735

[B8] Dennis CL Ross L Relationships among infant sleep patterns, maternal fatigue, and development of depressive symptomatology Birth 2005 32 187 193 16128972 10.1111/j.0730-7659.2005.00368.x

[B9] Miller AR Barr RG Eaton WO Crying and motor behavior of six-week-old infants and postpartum maternal mood Pediatrics 1993 92 551 558 8414826

[B10] Miller-Loncar C Bigsby R High P Infant colic and feeding difficulties Arch Dis Child 2004 89 908 912 15383432 10.1136/adc.2003.033233 PMC1719691

[B11] Smart J Hiscock H Early infant crying and sleeping problems: a pilot study of impact on parental well-being and parent-endorsed strategies for management J Paediatr Child Health 2007 43 284 290 17444831 10.1111/j.1440-1754.2007.01060.x

[B12] Miller AR Barr RG Infantile colic. Is it a gut issue? Pediatr Clin North Am 1991 38 1407 1423 1945549 10.1016/s0031-3955(16)38227-x

[B13] Treem WR Infant colic. A pediatric gastroenterologist’s perspective Pediatr Clin North Am 1994 41 1121 1138 7936776 10.1016/s0031-3955(16)38848-4

[B14] Lucassen PL Assendelft WJ Gubbels JW Effectiveness of treatments for infantile colic: systematic review BMJ 1998 316 1563 1569 9596593 10.1136/bmj.316.7144.1563 PMC28556

[B15] Shenassa ED Brown MJ Maternal smoking and infantile gastrointestinal dysregulation: the case of colic Pediatrics 2004 114 e497 e505 15466076 10.1542/peds.2004-1036

[B16] Lehtonen L Korvenranta H Eerola E Intestinal microflora in colicky and noncolicky infants: bacterial cultures and gas–liquid chromatography J Pediatr Gastroenterol Nutr 1994 19 310 314 7815263 10.1097/00005176-199410000-00009

[B17] Savino F Cresi F Pautasso S Intestinal microflora in breastfed colicky and non-colicky infants Acta Paediatr 2004 93 825 829 15244234

[B18] Savino F Bailo E Oggero R Bacterial counts of intestinal Lactobacillus species in infants with colic Pediatr Allergy Immunol 2005 16 72 75 15693915 10.1111/j.1399-3038.2005.00207.x

[B19] Savino F Cordisco L Tarasco V Molecular identification of coliform bacteria from colicky breastfed infants Acta Paediatr 2009 98 1582 1588 19604166 10.1111/j.1651-2227.2009.01419.x

[B20] Savino F Cordisco L Tarasco V Antagonistic effect of Lactobacillus strains against gas-producing coliforms isolated from colicky infants BMC Microbiol 2011 11 157 Doi: 10.1186/1471-2180-11-157 21718486 10.1186/1471-2180-11-157 PMC3224137

[B21] Aloisio I Santini C Biavati B Characterization of Bifidobacterium spp. strains for the treatment of enteric disorders in newborns Appl Microbiol Biotechnol 2012 96 1561 1576 22588500 10.1007/s00253-012-4138-5

[B22] Higgins JPT, Green S Cochrane Handbook for Systematic Reviews of Interventions Version 5.1.0 The Cochrane Collaboration 2011 Available from http://www.cochrane-handbook.org

[B23] Hozo SP Djulbegovic B Hozo I Estimating the mean and variance from the median, range, and the size of a sample BMC Med Res Methodol 2005 5 13 15840177 10.1186/1471-2288-5-13 PMC1097734

[B24] Savino F Pelle E Palumeri E Lactobacillus reuteri (American Type Culture Collection Strain 55730) versus simethicone in the treatment of infantile colic: a prospective randomized study Pediatrics 2007 119 e124 e130 17200238 10.1542/peds.2006-1222

[B25] Savino F Cordisco L Tarasco V Lactobacillus reuteri DSM 17938 in infantile colic: a randomized, double-blind, placebo-controlled trial Pediatrics 2010 126 e526 e533 20713478 10.1542/peds.2010-0433

[B26] Szajewska H Gyrczuk E Horvath A Lactobacillus reuteri DSM 17938 for the Management of Infantile Colic in Breastfed Infants: A Randomized, Double-Blind, Placebo-Controlled Trial J Pediatr 2013 162 257 262 22981952 10.1016/j.jpeds.2012.08.004

[B27] Egervärn M Danielsen M Roos S Lindmark H Lindgren S Antibiotic susceptibility profiles of Lactobacillus reuteri and Lactobacillus fermentum J Food Prot 2007 70 412 418 17340877 10.4315/0362-028x-70.2.412

[B28] Savino F Tarasco V New treatments for infant colic Curr Opin Pediatr 2010 22 791 797 20859207 10.1097/MOP.0b013e32833fac24

[B29] Rosander A Connolly E Roos S Removal of antibiotic resistance gene-carrying plasmids from Lactobacillus reuteri ATCC 55730 and characterization of the resulting daughter strain, L. reuteri DSM 17938 Appl Environ Microbiol 2008 74 6032 6040 18689509 10.1128/AEM.00991-08 PMC2565949

[B30] Lucas A St J-RI Crying, fussing and colic behaviour in breast- and bottle-fed infants Early Hum Dev 1998 53 9 18 10193923 10.1016/s0378-3782(98)00032-2

[B31] Rhoads JM Fatheree NY Norori J Altered fecal microflora and increased fecal calprotectin in infants with colic J Pediatr 2009 155 823 828 e1 19628216 10.1016/j.jpeds.2009.05.012

[B32] Ross JS Gross CP Krumholz HM Promoting transparency in pharmaceutical industry-sponsored research Am J Public Health 2012 102 72 80 22095335 10.2105/AJPH.2011.300187 PMC3319748

[B33] Dunn AG Gallego B Coiera E Industry influenced evidence production in collaborative research communities: a network analysis J Clin Epidemiol 2012 65 535 543 22300677 10.1016/j.jclinepi.2011.10.010

[B34] Spinler JK Taweechotipatr M Rognerud CL Human-derived probiotic Lactobacillus reuteri demonstrate antimicrobial activities targeting diverse enteric bacterial pathogens Anaerobe 2008 14 166 171 18396068 10.1016/j.anaerobe.2008.02.001 PMC2494590

[B35] Lin YP Thibodeaux CH Pena JA Probiotic Lactobacillus reuteri suppress proinflammatory cytokines via c-Jun Inflamm Bowel Dis 2008 14 1068 1083 18425802 10.1002/ibd.20448

[B36] Liu Y Fatheree NY Mangalat N Human-derived probiotics Lactobacillus reuteri strains differentially reduce intestinal inflammation Am J Physiol Gastrointest Liver Physiol 2010 299 G1087 G1096 20798357 10.1152/ajpgi.00124.2010 PMC2993169

[B37] Smits H Engering A van der Kleij D Selective probiotic bacteria induce IL-10-producing regulatory T cells in vitro by modulating dendritic cell function through dendritic cell-specific intercellular adhesion molecule 3-grabbing nonintegrin J Allergy Clin Immunol 2005 115 1260 1267 15940144 10.1016/j.jaci.2005.03.036

[B38] Sung V Hiscock H Tang M Probiotics to improve outcomes of colic in the community: Protocol for the Baby Biotics randomised controlled trial BMC Pediatr 2012 12 135 Doi: 10.1186/1471-2431-12-135 22928654 10.1186/1471-2431-12-135 PMC3508922

